# 2 μm passively mode-locked thulium-doped fiber lasers with Ta_2_AlC-deposited tapered and side-polished fibers

**DOI:** 10.1038/s41598-021-99928-z

**Published:** 2021-10-28

**Authors:** H. Ahmad, M. F. M. Azri, R. Ramli, M. Z. Samion, N. Yusoff, K. S. Lim

**Affiliations:** 1 Photonics Research Centre, Universiti Malaya, 50603 Kuala Lumpur, Malaysia; 2 Department of Physics, Faculty of Science, Universiti Malaya, 50603 Kuala Lumpur, Malaysia

**Keywords:** Optics and photonics, Physics

## Abstract

In this work, mode-locked thulium-doped fiber lasers operating in the 2 µm wavelength region were demonstrated using tantalum aluminum carbide (Ta_2_AlC)-based saturable absorbers (SAs) utilizing the evanescent wave interaction. The Ta_2_AlC MAX Phase was prepared by dissolving the Ta_2_AlC powder in isopropyl alcohol and then deposited onto three different evanescent field-based devices, which were the tapered fiber, side-polished fiber, and arc-shaped fiber. Flame-brushing and wheel-polishing techniques were used to fabricate the tapered and arc-shaped fibers, respectively, while the side-polished fiber was purchased commercially. All three SA devices generated stable mode-locked pulses at center wavelengths of 1937, 1931, and 1929 nm for the tapered, side-polished, and arc-shaped fibers. The frequency of the mode-locked pulses was 10.73 MHz for the tapered fiber, 9.58 MHz for the side-polished fiber, and 10.16 MHz for the arc-shaped fiber. The measured pulse widths were 1.678, 1.734, and 1.817 ps for each of the three SA devices. The long-term stability of the mode-locked lasers was tested for each configuration over a 2-h duration. The lasers also showed little to no fluctuations in the center wavelengths and the peak optical intensities, demonstrating a reliable, ultrafast laser system.

## Introduction

The discovery of optical fibers by Charles Kao and George A. Hockam in 1966 gave the inroads for optical amplifier development with a wavelength range of 1.46–1.53 µm^1,2^, 1.53–1.565 µm^3,4^ and 1.565–1.625 µm^5–10^. Consequently, the development of laser configuration such as pulsed^11,12^, dual and multiwavelength^13–22^, and optical sensors^23–25^ has been the focus of many research laboratories. Although there have been numerous works on pulsed lasers operating at wavelengths of 1 μm and 1.5 μm, there is an increasing interest to generate short pulses in the 2 μm wavelength region for several applications such as in spectroscopy^26^, gas detection^27^, laser ablation^28^, light detection and ranging (LIDAR) for remote sensing^29^, plastic and glass processing^30^ as well as in the medical field^31^. Lasing in the 2 µm is commonly achieved using thulium-doped fibers (TDFs) as the gain medium, as TDFs have a broad amplification range of 400 nm, ranging from 1700 to 2100 nm^32^. The 2 µm wavelength region is also of interest as it coincides with the absorption lines of water (H_2_O) and several leading greenhouse gases such as carbon dioxide (CO_2_) and nitrogen dioxide (NO_2_)^33^. Although lasing in the 2 µm region has traditionally been demonstrated with continuous wave (CW) outputs, recent advances in fiber laser technologies have increased the development of 2 µm pulsed fiber lasers that can generate short pulses with pulse durations in the pico- or femtosecond range.

Pulsed laser generation can be achieved either by Q-switching or mode-locking. In the former, short pulses with high output energies can be produced using an optical component incorporated in the laser cavity to modulate the Q-factor. In the latter, the oscillating longitudinal modes present in the laser cavity are phase locked when an optical component is introduced in the optical cavity. Pulse generation in fiber lasers could be obtained using two main techniques; namely active and passive^34^. Active techniques require the use of external modulators such as acousto-optic and electro-optic modulators^35^. It, however, causes the system to be bulky and inflexible due to the extra electronic components needed to be used. In comparison, a passive technique allows for the development of a more compact and versatile system. Saturable absorbers (SAs) are used to saturate the molecules or atoms, whereby the optical absorption decreases as the light intensity increases. Due to this nonlinear optical response of SAs together with a narrow optical bandgap, a high damage threshold, and a wide bandwidth, SAs are suitable devices to generate short pulses using the Q-switching and mode-locking techniques. SAs can be divided into two groups, namely artificial SAs and real SAs. Nonlinear optical loop mirrors (NOLMs), nonlinear amplification loop mirrors (NALMs), or nonlinear polarization evolution (NPE) are examples of artificial SAs. Artificial SAs are not suitable for commercialization due to its sensitivity to environmental changes and large size despite their positive attributes of near-instantaneous response time and high modulation depth^36–39^. Semiconductors saturable absorbers mirrors (SESAM), a real SA, were chosen as the SA of choice for nonlinear absorption property that depends on light intensity^40,41^. However, the disadvantages of SESAM are the operating bandwidth is narrow, complex design, costly, and has a low-damage threshold^42^.

In view of those limitations mentioned above, new SA nanomaterials are now the main focus of research in ultrafast laser worldwide. To date, various kinds of materials that exhibit intensity-dependent transmission have been used as SAs, namely graphene^43^, carbon nanotubes (CNTs)^44^, black phosphorus (BP)^45^, transition metal dichalcogenides (TMDs)^46,47^, metal–organic frameworks (MOF)^48^, transition metal oxides^49^ and topological insulators (TIs)^50^. Recently, a new type of material named MXenes has been widely explored for various optoelectronic applications due to their unique optical properties^51,52^. It also makes them a great candidate to be used as SAs in generating ultrafast lasers. MXenes are typically obtained from their precursor, the MAX phases^53^. Compared to its counterpart, MAX phases are favorable as it does not require the use of strong etching solutions that contain fluoride ions (F^−^) such as hydrofluoric acid (HF), ammonium bifluoride (NH_4_HF_2_), or a mixture of hydrochloric acid (HCl) and lithium fluoride (LiF), thus minimizing the fabrication process and cost^54^. The MAX phases are also useful for high-temperature applications as they comprise of ternary transition-metal carbides that have metal and ceramic properties. This makes them to have a good thermal and electrical conductivity, as well as having a high damage threshold^55^. Several works demonstrate the use of MAX phases as SAs in generating pulses in fiber lasers. For instance, Lee et al. demonstrated the use of a titanium aluminum carbide (Ti_2_AlC) SA to generate Q-switched pulses with a maximum pulse energy of 22.58 nJ in an erbium-doped fiber laser (EDFL) cavity^56^. Jafry et al. also demonstrated an ultrashort pulse generation in an EDFL cavity using a Ti_3_AlC_2_-PVA SA. The generated mode-locked pulses had a pulse width of 3.68 ps wavelength of 1577 nm^57^. These demonstrations show the tremendous potential of MAX phases in generating short pulses in fiber lasers, which would allow further exploration of various MAX phases with other combination of the early transition metals.

In addition to these aforementioned SA materials, the structure of the SA devices has a significant impact on the SA performance. SA materials could be prepared and integrated into fiber laser cavities by several arrangements, which are typically done using the optical deposition method onto fiber ferules^58,59^ or substrates and polymer hosts^60,61^. Although these methods allow for a more direct integration, they limit the operation of the lasers at low power due to their poor heat dissipation and low optical damage thresholds. Another method that is attracting a great interest of late is the SA devices that utilize the nonlinear interaction of the propagating light in the optical fiber with the nonlinear materials. Since the light-matter interaction is realized via the evanescent field, this approach is more efficient. It can provide better functionality by eliminating the issue of heat accumulation. Various types of saturable absorber devices have been used as to generate pulses in fiber lasers. For example, Mouchel et al. utilized a graphene-coated tapered fiber to generate mode-locked pulses in an Er:Yb doped double-clad fiber laser with a high average output power of 520 mW when being pumped to a maximum pump power of 5 W^62^. Zhou et al. obtained a mode-locked laser using a lead sulfide (PbS) being deposited onto an arc-shaped fiber, in which the SA had a damage threshold of higher than 3.5 kW^63^. Other reports have also shown the use of side-polished fibers (SPFs) and nonlinear materials to generate mode-locked pulses. Li et al. reported a harmonic mode-locking at 1879 nm using a graphene-deposited SPF as the SA, where up to the 21st harmonics were obtained at a pump power of 500 mW^64^. Khazaeinezhad et al. integrated a molybdenum disulfide (MoS_2_)-SPF in an EDFL cavity to get mode-locked fiber lasers at both anomalous and normal-dispersion regimes, with the mode-locking operating being sustained at a pump power of more than 600 mW^65^. Liu et al.^66^ deposited SnSe_2_ onto a tapered fiber to produce pulses at the region of 1550 mm with a repetition rate of 8.3 MHz. A demonstration of tapered fiber coated with Ag_2_S for the generation of ultrafast laser was shown by Feng et al.^67^ with values of pulse width and signal-to-noise ratio of 980 fs and 73 dB, respectively. These demonstrations have shown that the use of SA devices that utilize the evanescent field interaction would be suitable in developing robust ultrafast laser systems to suit the demand for future high-power laser systems.

In this work, we explored three different types of SA devices: the tapered fiber, the side-polished fiber, and the arc-shaped fiber to generate mode-locked pulses in the 2 µm wavelength region. A Ta_2_AlC MAX phase was first prepared in the solution form by ultrasonication Ta_2_AlC powder in isopropyl alcohol (IPA), deposited onto the three fibers. The MAX phase was composed of tantalum (Ta) as the early transition metal instead of the common titanium (Ti). Each of the SA devices was then individually inserted into a TDFL cavity to generate mode-locked pulses with frequencies between 9 and 11 MHz and pulse widths between 1.678 and 1.817 ps. The pump power of the laser cavity could be increased up to 1 W with all three devices, maintaining the mode-locking operation in the 2 µm without any damage to the SA devices. The results show the role of the SA devices as promising and robust SA devices for the development of high-power fiber lasers.

## Characterization of the Ta_2_AlC MAX phase solution

**Figure 1:**
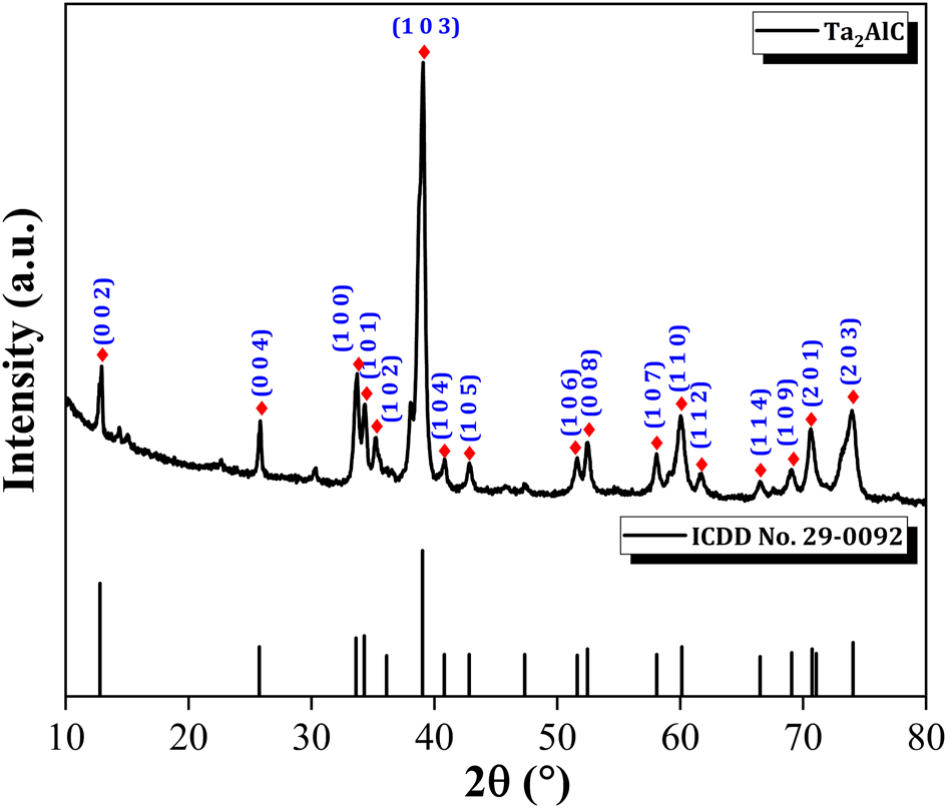
XRD pattern of the Ta_2_AlC MAX Phase.

The crystalline phase of the Ta_2_AlC MAX Phase was first assessed using X-ray powder diffraction (XRD). The measurement was recorded using a Malvern Panalytical Empyrean XRD by utilizing Cu–Kα radiation (λ = 0.1541 nm) as the X-ray source and by scanning in the 2θ range of 10°–80°. The obtained XRD spectrum is shown in Fig. 1.

It was observed from the XRD pattern that a distinct prominent peak at 2θ = 39.07° together with a few sharp peaks was detected at 2θ = 12.92°, 25.84°, 33.72°, 34.34°, 35.25°, 39.07°, 40.81°, 42.84°, 51.63°, 52.46°, 58.08°, 60.06°, 61.72°, 66.48°, 69.06°, 70.61°, and 73.96°. These peaks were assigned to the (1 0 3), (0 0 2), (0 0 4), (1 0 0), (1 0 1), (1 0 2), (1 0 4), (1 0 5), (1 0 6), (0 0 8), (1 0 7), (1 1 0), (1 1 2), (1 1 4), (1 0 9), (2 0 1) and (2 0 3) planes of the Ta_2_AlC MAX Phase, respectively. The result was in accordance with the ICDD card No. 29-0092 for Ta_2_AlC^68^ as has been plotted in Fig. 1 for comparison and is similar with previous works^69,70^. The absence of other impurity peaks indicates the purity of the sample, and the high crystallinity of the sample was proven by the presence of sharp diffraction peaks in the XRD pattern of the Ta_2_AlC MAX Phase.

**Figure 2:**
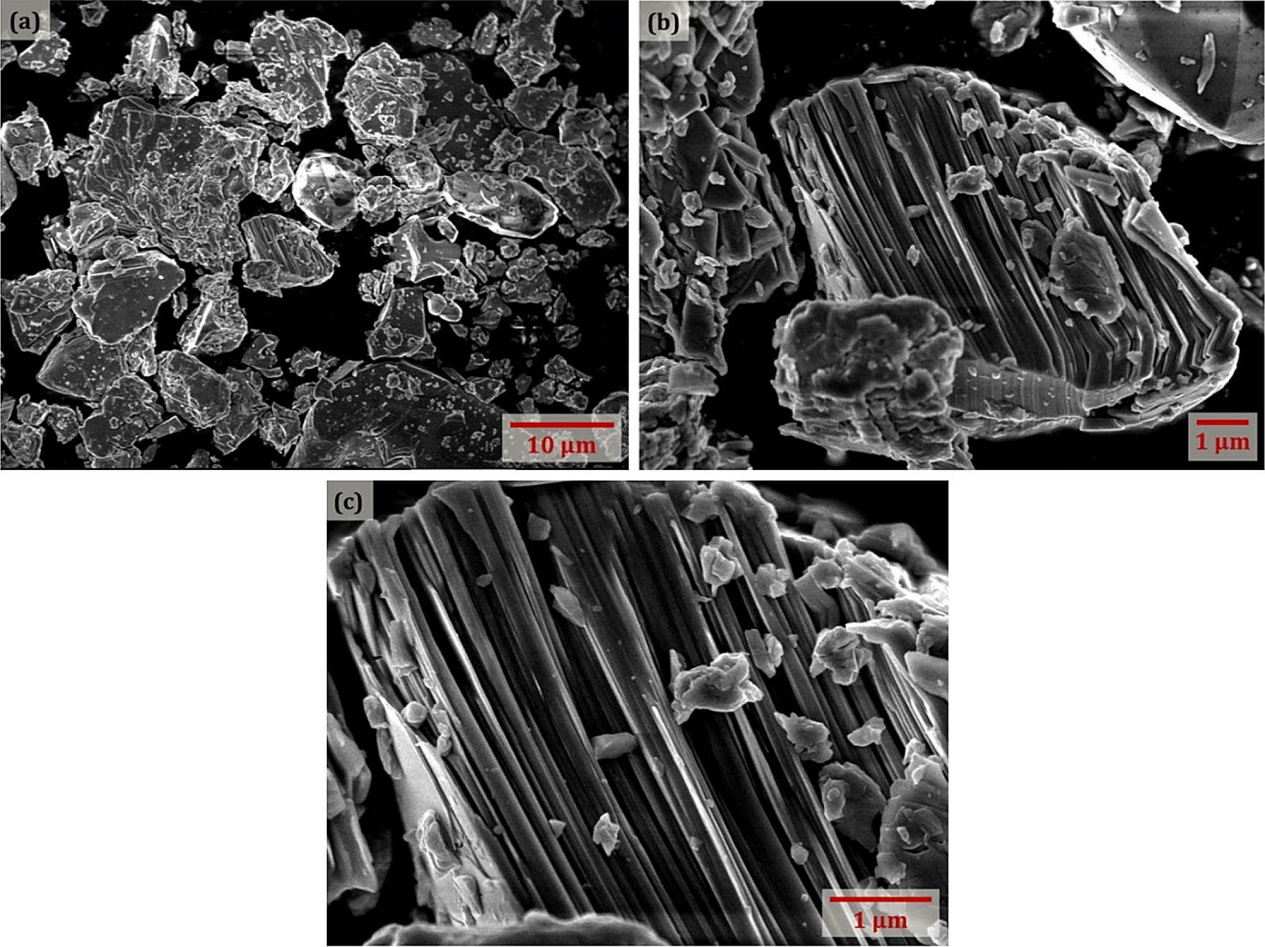
FESEM images of Ta_2_AlC MAX Phase captured at (**a**) 5k, (**b**) 10k, and (**c**) 20k× magnification with 5.0 kV of operating voltage.

The morphology of the Ta_2_AlC MAX Phase was then examined using a JEOL JSM 7600-F field emission electron microscope (FESEM) at an accelerating voltage of 5.0 kV. The FESEM images captured at various magnifications are shown in Fig. 2. The Ta_2_AlC MAX Phase had a flake-like structure with irregular shapes and had different lateral flake sizes varying from about 0.5–18 μm, as shown in Fig. 2a. The single grain of Ta_2_AlC in Fig. 2b indicates that the Ta_2_AlC consisted of assembled Ta–C layers and Al layers that are alternately stacked to form the Ta_2_AlC. At the highest magnification shown in Fig. 2c, it is seen that the layers are densely packed and that a single layer of the Ta_2_AlC had an average thickness of < 100 nm.

**Figure 3:**
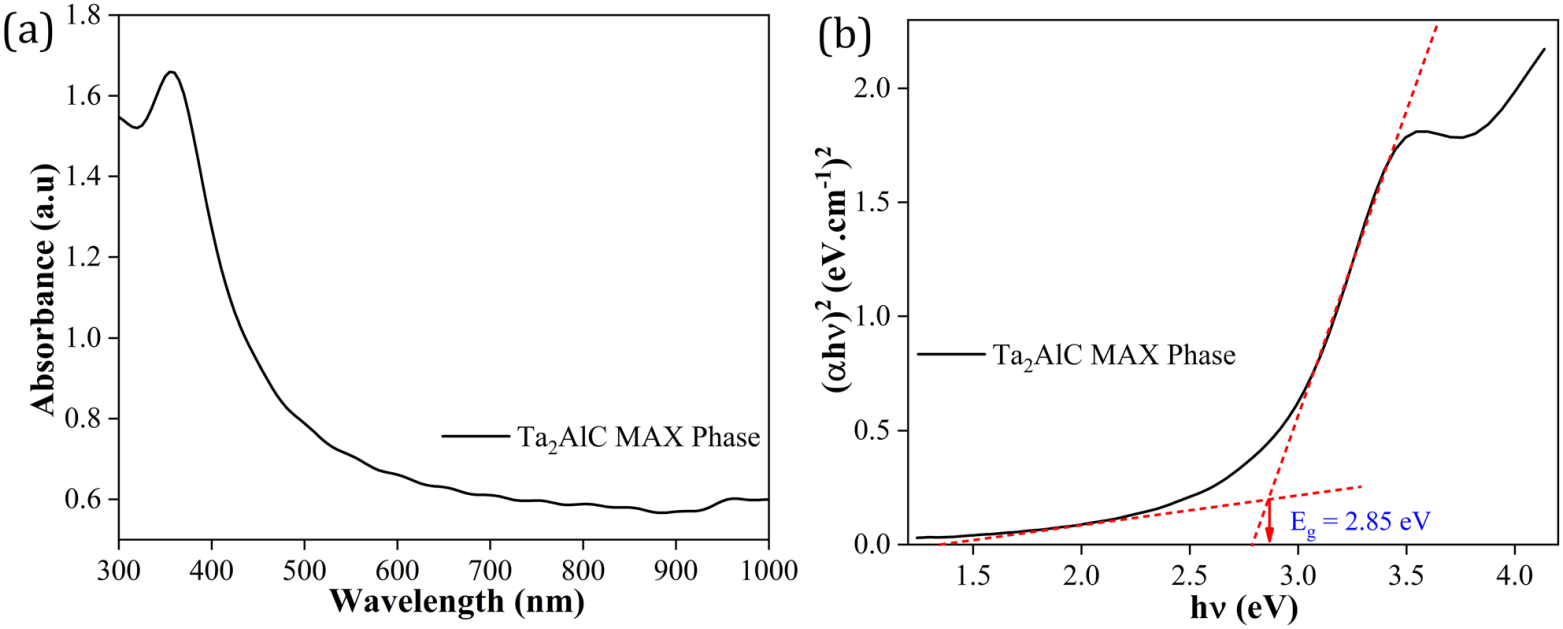
(**a**) UV–Vis absorbance spectrum of Ta_2_AlC MAX Phase and (**b**) corresponding Tauc plot for bandgap measurement.

The Ta_2_AlC MAX Phase was then tested using an Agilent Technologies Varian Cary 50 UV–Vis spectrometer to examine the absorption spectrum of the sample. Figure 3a depicts the UV–Vis absorption spectrum of Ta_2_AlC MAX Phase, where it is seen that Ta_2_AlC MAX Phase had an absorption peak at around 360 nm. The corresponding Tauc plot was presented in Fig. 3b and the bandgap (*E*_g_) was calculated using the Tauc relation, which was given by the following equation:

1 $$\alpha {\text{hv}} = {\text{A}}({\text{hv}} - E_{g} )^{n}$$

where α is absorption coefficient, A is a constant, h is Planck’s constant, ν is the frequency, *E*_g_ is the average band gap and n depends on the type of transition (for this sample, n = 1/2). The linear extrapolation to the Tauc plot was used to determine the bandgap of Ta_2_AlC MAX Phase which give the value of ~ 2.85 eV.

## Fabrication and characterization of the Ta_2_AlC-deposited tapered, arc-shaped and side polished fibers

### Ta_2_AlC-deposited tapered fiber

**Figure 4:**
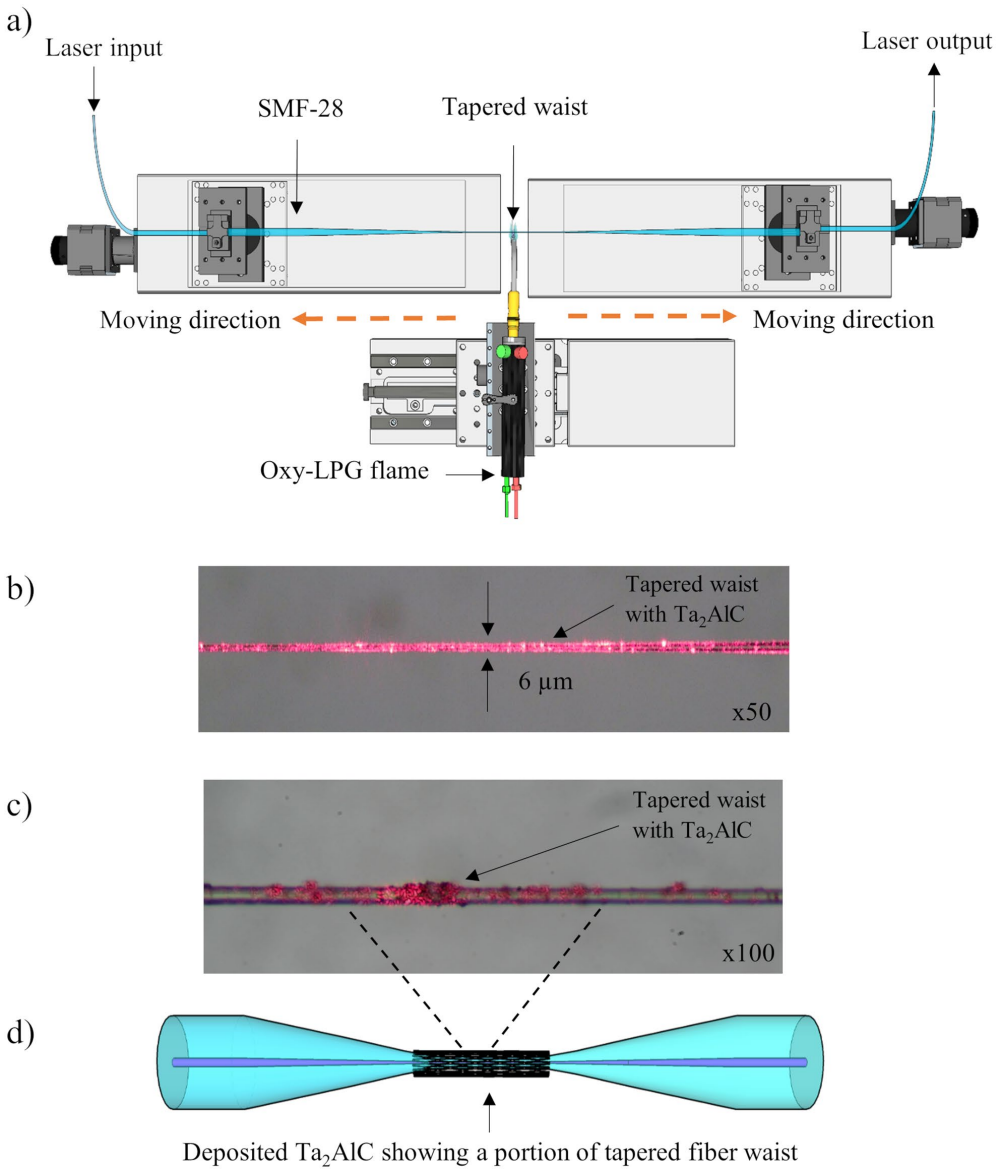
(**a**) The setup of flame brushing method to fabricate tapered fiber. (**b**) The microscopic image of the tapered fiber at ×50 and (**c**) ×100 magnification with the material. (**d**) The schematic drawing of the coated Ta_2_AlC tapered fiber (**a**) was drawn using SketchUp Make 2017 (Basic), Software Version: Windows 64-bit 17.2.2555. Available at https://www.sketchup.com/download/all.

The tapered fiber was fabricated using the flame brushing method, as shown in Fig. 4a. An approximately 6 cm-long bare fiber was first stripped and then fixed onto Newport FCL100 translation stages using fiber holders. The stages were used to pull and stretch the fiber after it has been softened using oxy-LPG flames so that the width of the optical fiber was reduced from a diameter of 125 μm to 6 μm, with a tapered length of about 2.5 cm. Upon completion, the insertion loss of the tapered fiber was measured using a light source (LS) and an optical power meter (OPM), giving a value of approximately 4.13 dB at 2000 nm.

The Ta_2_AlC in the solution form was then deposited onto the tapered fiber using the drop-cast method and was left until dry at room temperature. The insertion loss of the Ta_2_AlC-deposited tapered fiber was measured to be 4.63 dB. The microscopic image of the fabricated tapered fiber taken at 50 times magnification is shown in Fig. 4b, where the tapered waist was reduced to only 6 μm. In Fig. 4c, the microscopic image of the tapered fiber after the deposition of the Ta_2_AlC was also taken at 100 times magnification. It shows that the Ta_2_AlC was successfully coated around the waist of the fabricated tapered fiber. The graphical illustration of the MAX phase Ta_2_AlC-coated tapered fiber is also given in Fig. 4d.

**Figure 5:**
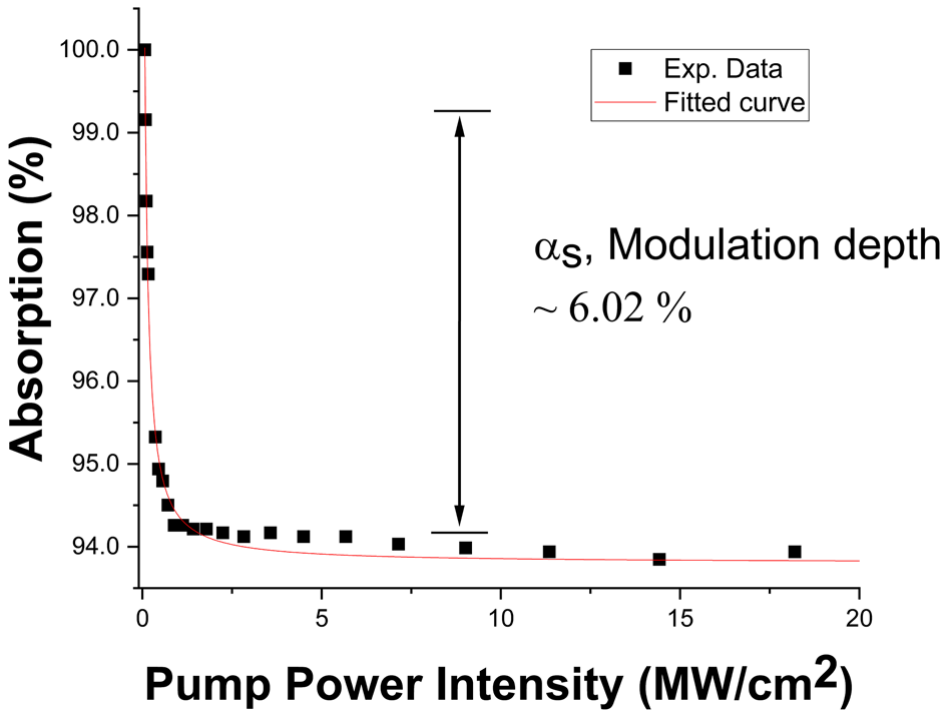
The nonlinear absorption measurement of the Ta_2_AlC-based tapered fiber.

The twin detector measurement technique^71^ was used to evaluate the nonlinear optical absorption properties of the Ta_2_AlC-deposited tapered fiber. The pulsed laser for the measurement was a FemtoFErb 1950 nm femtosecond fiber laser, from Toptica Photonics, with a pulse width of 90 fs and a repetition rate of 30 MHz. To calculate the modulation depth, the non-saturable absorption and the saturation intensity of the Ta_2_AlC-deposited tapered fiber, the experimental data obtained were fitted using the typical two-level saturation absorption model, which can be expressed as follows^72^:

2 $$\alpha \left( I \right) = \frac{{\alpha_{s} }}{{1 + I/I_{sat} }} + \alpha_{ns}$$

where *I* is the input intensity, *I*_*sat*_ is the saturation intensity and *α*_*ns*_ is the non-saturable absorption. For the Ta_2_AlC-deposited tapered fiber, the computed modulation depth and the saturation intensity as measured in Fig. 5 were 6.02% and 0.36 MW/cm^2^, respectively.

#### Ta_2_AlC-deposited side-polished fiber

**Figure 6:**
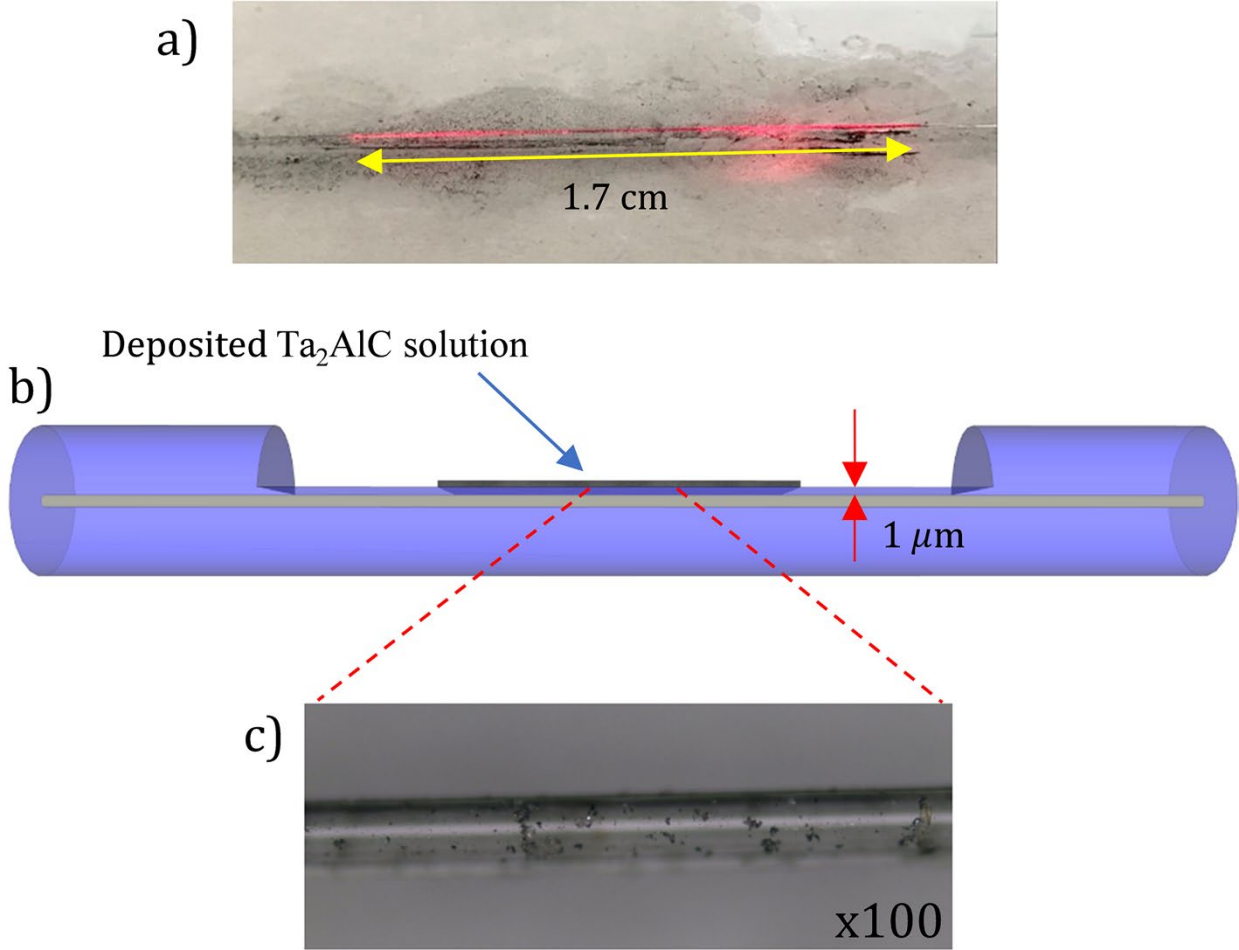
(**a**) The evanescent field at the polished region using a red-light source. (**b**) The deposited Ta_2_AlC on the SPF. (**c**) Microscopic image of the polished part of the side polished fiber at ×100 magnification.

**Figure 7:**
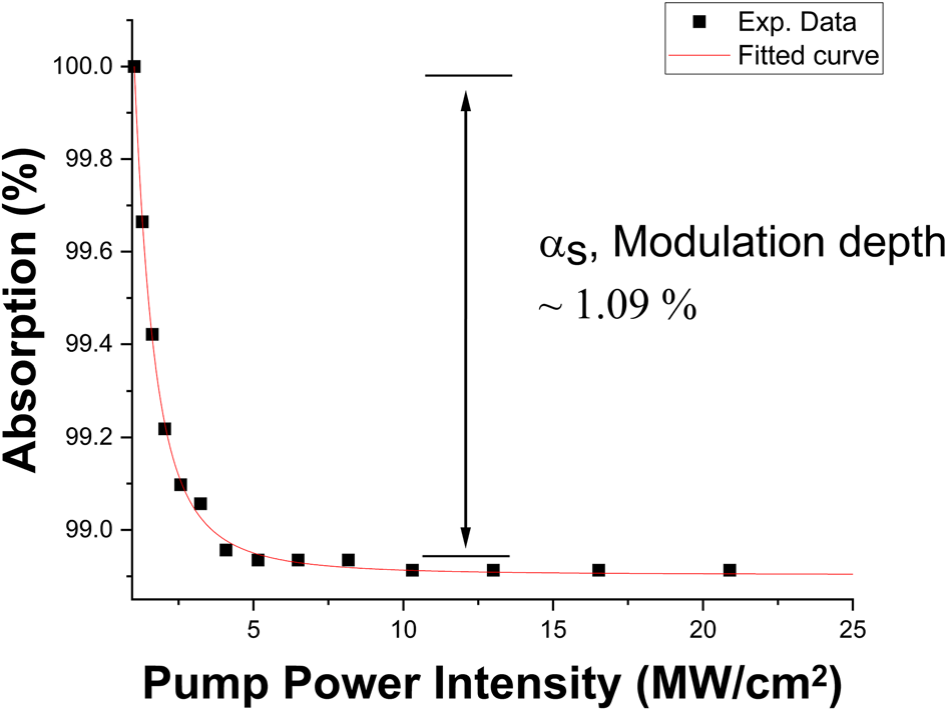
The nonlinear absorption test of the side polished fiber Ta_2_AlC.

The side-polished fiber (SPF) was commercially obtained from Phoenix Photonics. The polishing depth of the SPF was approximately 1 µm from the edge of the core, and the polished length was 1.7 cm, as given by the manufacturer. The insertion loss of the SPF without the Ta_2_AlC was measured to be 4.12 dB at 2000 nm. The Ta_2_AlC was drop-casted onto the polished region of the SPF, similarly as it was with the tapered fiber. The measured insertion loss of the SPF after the deposition of Ta_2_AlC was 4.80 dB. Figure 6a shows the image of the Ta_2_AlC-deposited SPF when being injected with a red-light source. It is observed that scattered light was seen along the polished region of the SPF, confirming that the polished length was approximately 1.7 cm. The deposited Ta_2_AlC at the polished part of the SPF is illustrated in Fig. 6b, while the microscopic image of the deposited polished region of the SPF at 100 times magnification is shown in Fig. 6c. The nonlinear absorption test for the Ta_2_AlC-deposited side polished fiber was conducted using the twin detection measurement method as described previously. From Fig. 7, the modulation depth and the saturation intensity were calculated to be 1.09% and 1.63 MW/cm^2^ for the Ta_2_AlC-deposited SPF.

#### Ta_2_AlC-deposited arc-shaped fiber

**Figure 8:**
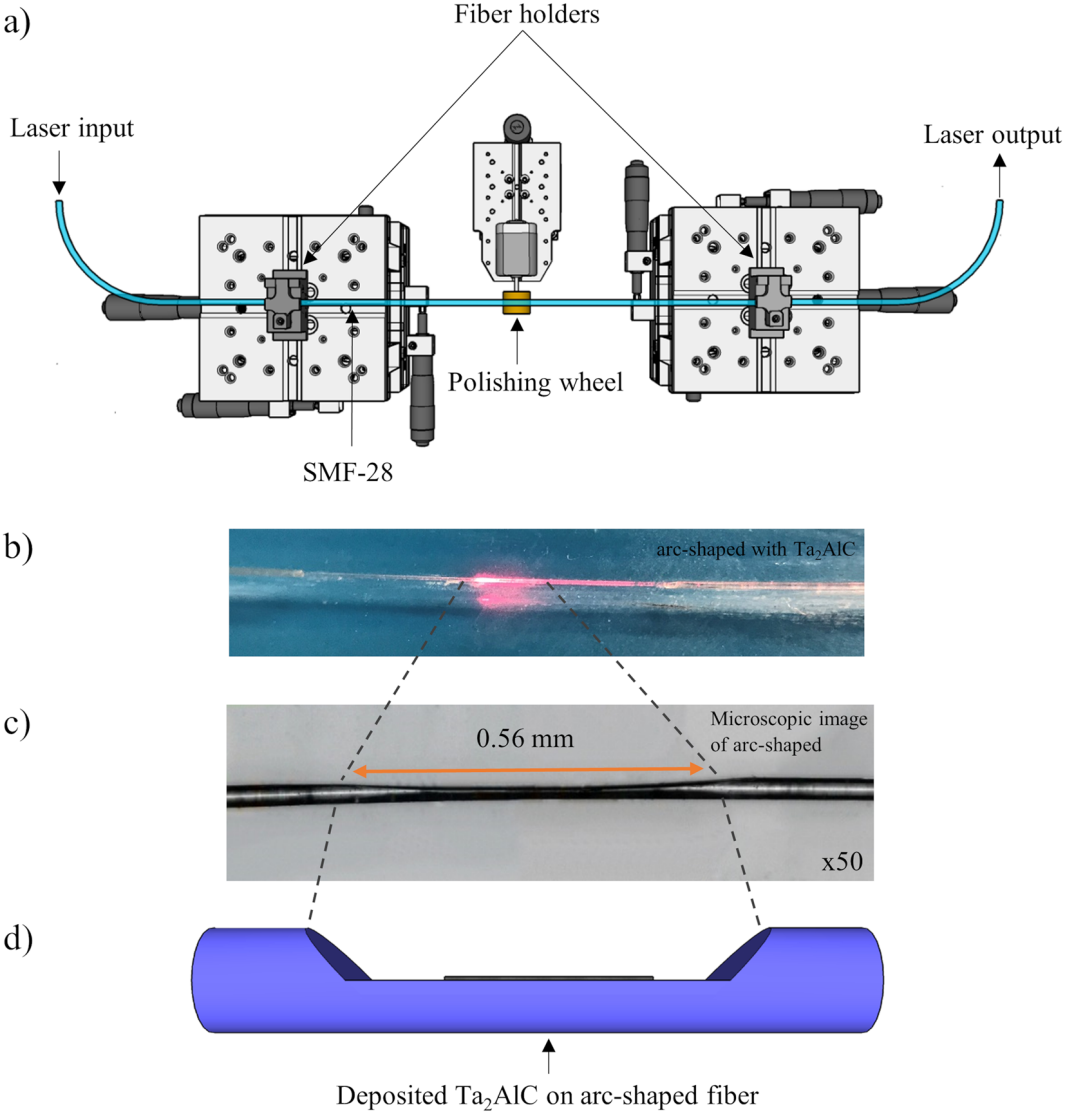
(**a**) Schematic illustration of wheel polishing method to fabricate the arc-shaped fiber. (**b**) The interaction of the arc-shaped fiber embedded with the Ta_2_AlC nanoparticles when a red-light source was injected. (**c**) The microscopic image of the side profile of the arc-shaped fiber at × 50 magnification. (**d**) The illustration of the deposited Ta2AlC onto the arc-shaped fiber. (**a**) Drawn using SketchUp Make 2017 (Basic), Software Version: Windows 64-bit 17.2.2555, Available at https://www.sketchup.com/download/all.

**Figure 9:**
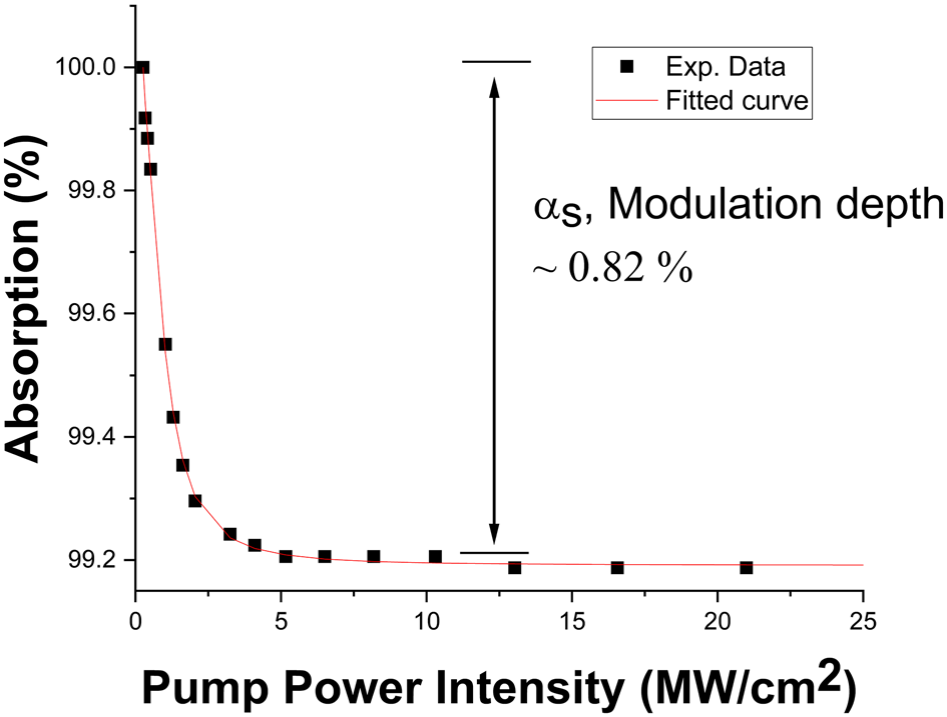
The nonlinear absorption test of the Ta_2_AlC-deposited arc-shaped fiber.

The arc-shaped fiber was fabricated using the polishing wheel method^73^, schematically shown in Fig. 8a. Two fiber holders and two mechanical alignment stages were used to hold a single-mode fiber (SMF-28) above a polishing wheel with a diameter of approximately 1.5 cm. The polishing wheel was wrapped with P800 grit sandpaper and then fixed on a motor shaft. The wheel was rotated and gradually raised onto the bottom side of the SMF. The polishing process was carried out while the insertion loss of the arc-shaped fiber was simultaneously monitored by connecting one end of the fiber to a light source (LS) and the other to an optical power meter (OPM). The polishing process was stopped when the insertion loss of the arc-shaped fiber was measured to be approximately 4.20 dB. After the process was completed, the arc-shaped fiber was carefully transferred onto a glass slide. The deposition of the Ta_2_AlC solution onto the arc-shaped fiber was carried out in the same manner as described in the previous section. The measured insertion loss arc-shaped fiber after the deposition of Ta_2_AlC was 4.75 dB. The polarization dependent losses (PDLs) of the Ta_2_AlC-deposited arc-shaped fiber and the SPF were also measured as their asymmetrical fiber structure could possibly induce the polarization effect. However, it is found that ther PDL values of the Ta_2_AlC-deposited arc-shaped and side-polished fibers were less than 1 dB, which were too low to be a major contributor in the mode-locked laser generation. This was also investigated by Jung et al.^74^. When injected with a red-light source, scattered light was observed in the polished region of the arc-shaped fiber, as shown in Fig. 8b. The microscopic image of the arc-shaped fiber's side profile at 50 times magnification, as shown in Fig. 8c, d, illustrates the deposited Ta_2_AlC onto the arc-shaped fiber. The polished length and the polishing depth of the arc-shaped fiber was determined to be 0.56 mm and approximately 55 µm, respectively. Therefore, the polishing height from the edge of the core for the arc-shaped fiber was approximately 3 µm. It is also seen from the figure that the polished region has an arc-shaped, while a side-polished fiber has a more abrupt reduction in its polished area^75^. Figure 9 shows the nonlinear absorption plot of the Ta_2_AlC-deposited arc-shaped fiber. From the graph, the modulation depth and the saturation intensity were calculated to be 0.82% and 1.03 MW/cm^2^, respectively.

The modulation depths and the saturation intensities of the Ta_2_AlC-based SA devices are summarized in Table 1. The Ta_2_AlC-deposited tapered fiber had the lowest saturation intensity of 0.36 MW/cm^2^, with a modulation depth of 6.02%. Although the Ta_2_AlC-deposited SPF and arc-shaped fiber had lower modulation depth at 1.09% and 0.82%, the modulation depth of 6.02% was still capable of generating mode-locked pulses. Previous works have been reported using SAs with modulation depths of above 5%^76–78^. It was expected that Ta_2_AlC-deposited tapered fiber would have the lowest mode-locking threshold, as it had the lowest saturation intensity compared to the 1.63% of the Ta_2_AlC-deposited SPF and 1.03% of the Ta_2_AlC-deposited arc-shaped fiber. A low saturation intensity would allow the SA to be saturated at a lower pump power to induce the mode-locking operation^79^.

**Table 1:** Summary of the nonlinear properties of each of the SA devices.

SA Device	Modulation depth (%)	Saturation intensity (MW/cm^2^)
Ta_2_AlC-deposited tapered fiber	6.02	0.36
Ta_2_AlC-deposited SPF	1.09	1.63
Ta_2_AlC-deposited arc-shaped fiber	0.82	1.03

## Experimental setup

**Figure 10:**
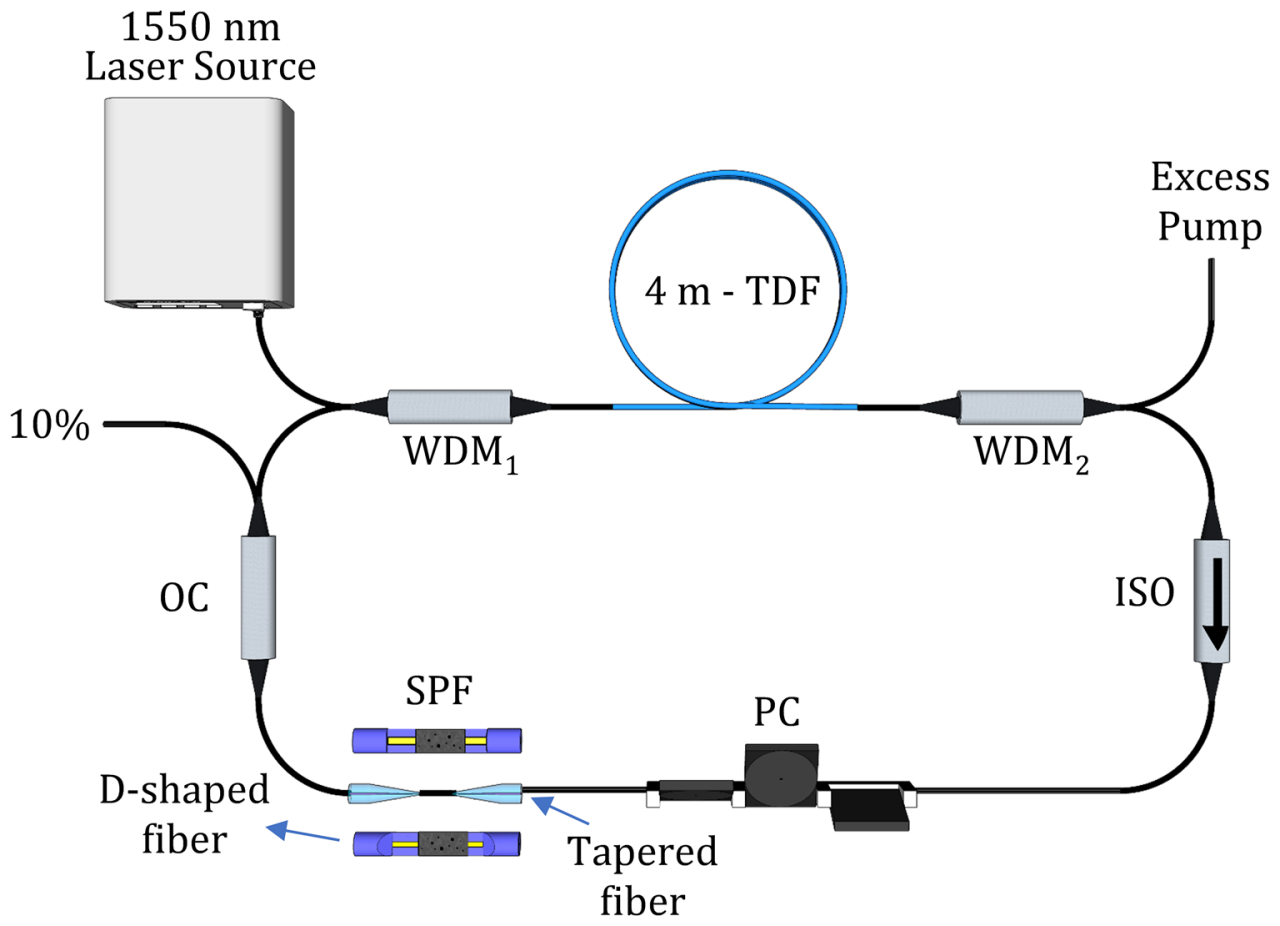
Illustration of the experimental setup for the 2 µm mode-locked Thulium-doped fiber laser (TDFL). This figure was drawn using SketchUp Make 2017 (Basic), Software Version: Windows 64-bit 17.2.2555. Available at https://www.sketchup.com/download/all.

The experimental setup for the 2 µm mode-locked thulium-doped fiber laser (TDFL) using the Ta_2_AlC-based SAs is presented in Fig. 10. A 1565 nm laser source (LS) with a maximum output power of 1 W was used to pump the gain medium through the 1550 nm port of the 1550/2000 nm wavelength division multiplexer (WDM). The gain medium was a 4-m long TmDF200 thulium-doped fiber (TDF) from OFS Inc, connected to the common port of WDM_1_. The absorption coefficient of the TDF was 22 dB/m at 1565 nm, and the core and cladding diameters of the TDF were 9 and 125 µm, respectively. The TDF was then connected to the common port of WDM_2_, where the 1550 nm port was used to remove any excess pump. The 2000 nm port of WDM_2_ was connected to the input port of a 2000 nm isolator to obtain a unidirectional operation in the laser cavity. A polarization controller (PC) was connected to the isolator's output to adjust the propagating signal's polarization state. The Ta_2_AlC-based SA, either in tapered fiber, a side-polished fiber, or an arc-shaped fiber, was connected to the other end of the PC. The cavity was completed by connecting the other end of the SA device to a 90:10 coupler, where 90% of the signal was circulated back into the cavity, and another 10% was taken as the laser output.

Since there were three different types of SA devices used in this experiment, the total length for each fiber was slightly different, resulting in a different net cavity dispersion for each setup. Table 2 gives the calculated group velocity dispersion (GVD) values for each SA device at their respective operating wavelengths. The manufacturer provided the GVDs of the TmDF200, while the GVDs of SMF-28 were estimated from Corning dispersion equation.

**Table 2:** The computed dispersion values for the TDFL cavities with different types of SA devices.

Device	Central wavelength (nm)	GVD TmDF 200 (ps^2^/m)	TmDF200 length (m)	GVD SMF-28 (ps^2^/m)	SMF-28 length (m)	Total cavity length (m)	Net dispersion (ps^2^)
Tapered	1937	− 0.214	4	− 0.0649	15.35	19.35	− 1.85
SPF	1931	− 0.211	4	− 0.0645	17.81	21.81	− 1.99
Arc-shaped	1929	− 0.208	4	− 0.0643	16.43	20.43	− 1.88

The net cavity dispersion for each of the mode-locked laser was computed by ${Dispersion}_{net}={GVD}_{TmDF200}{Length}_{TmDF200}+ {GVD}_{SMF}{Length}_{SMF}$. From the values given in Table 2, the calculated net cavity dispersions for the mode-locked lasers were − 1.85, − 1.99, and − 1.88 ps^2^ for the cavity with the tapered fiber, SPF, and arc-shaped fiber, respectively. It indicates that all the mode-locked lasers operated in the anomalous dispersion regime.

## Results and discussion

The experiment was first conducted without any of the SA devices to observe the operation of the fiber laser. As expected, the TDFL without any SA only operated in the continuous wave (CW) regime as no pulses were observed in the oscilloscope as the pump power was increased to a maximum of 1 W. The experiment was then continued by integrating the Ta_2_AlC-deposited tapered fiber into the laser cavity. With fine-tuning of the PC, the fundamental mode-locking of the TDFL was observed at a threshold pump power of 245 mW. The fundamental mode-locking could be obtained until the pump power reached 480 mW. As the pump power was further increased until the maximum, the mode-locked operation was sustained but the laser operated at higher harmonics. As our interest was mainly on the fundamental operation of the mode-locked laser, the laser characteristics were only recorded when the pulsed laser operated at the fundamental frequency.

**Figure 11:**
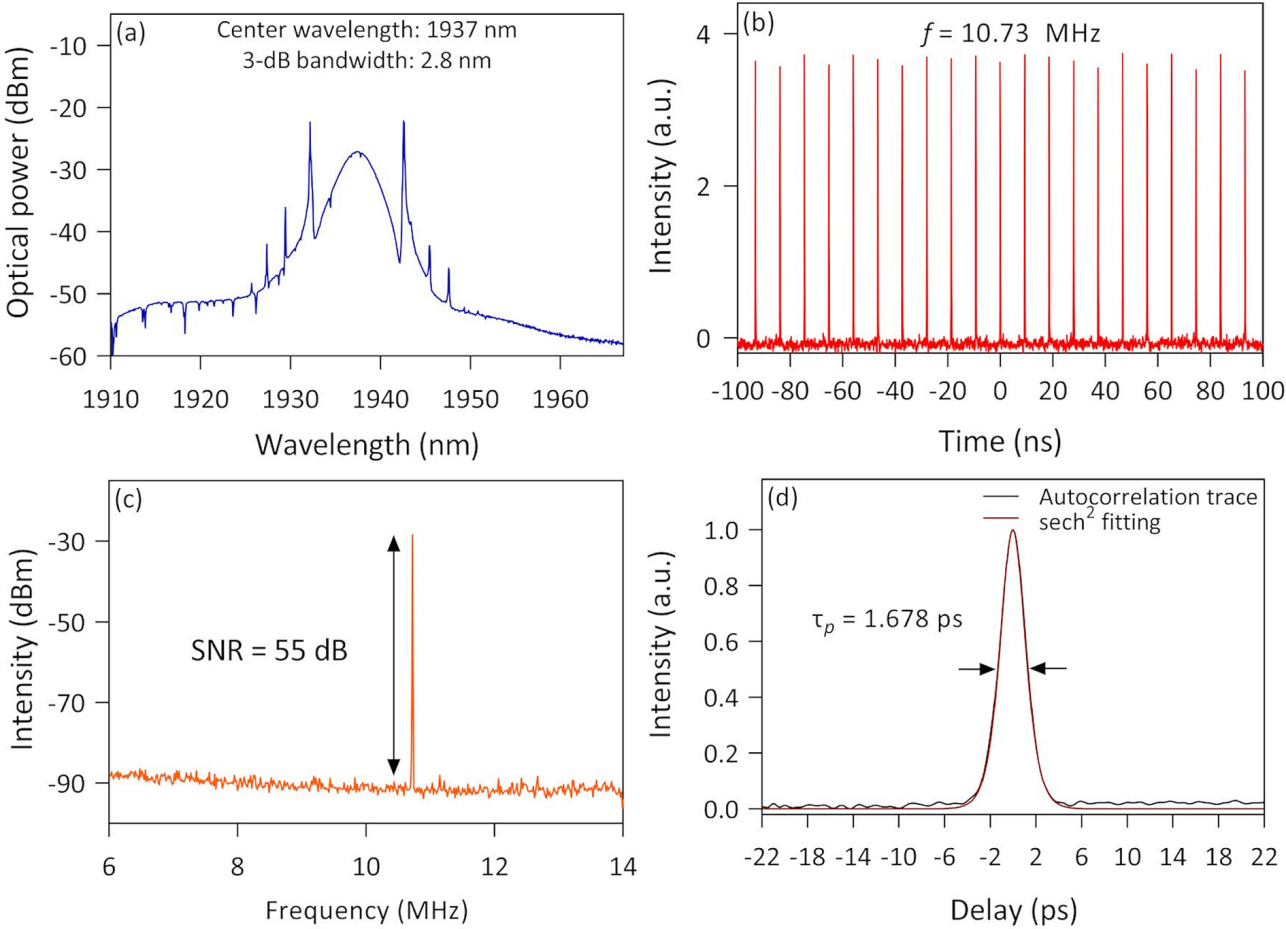
The output characteristic for mode-locked thulium-doped fiber laser using the Ta_2_AlC tapered fiber saturable absorber (**a**) optical spectrum, (**b**) pulse train, (**c**) radio frequency spectrum, and (**d**) autocorrelation trace.

The characteristics of the mode-locked laser using the Ta_2_AlC-deposited tapered fiber are shown in Fig. 10. The optical spectrum recorded in Fig. 11a shows a broad laser spectrum at a center wavelength of 1937 nm, having a 3-dB bandwidth of 2.8 nm. It was apparent that Kelly's sidebands were observed in the soliton spectrum when the mode-locking operation was initiated, which was expected as the mode-locked TDFL was operating in the anomalous dispersion regime. From the values obtained from the optical spectrum, the transform-limited pulse width could be estimated by the equation:

3 $${\tau }_{p}=\frac{TBP\times {{(\lambda }_{c})}^{2}}{c \times \Delta \lambda }$$

where c is the speed of light, TBP is the time-bandwidth product, λ_c_ is the center wavelength, and ∆λ is the 3-dB bandwidth. Taking the TBP to be 0.315 for a sech^2^ pulse profile, the center wavelength to be 1937 nm, and the 3-dB bandwidth to be 2.8 nm, the transform-limited pulse width was calculated to be 1.407 ps. Figure 11b shows that the oscilloscope trace of the mode-locked pulses had a repetition rate of 10.73 MHz, which correlates with the cavity round trip time estimated from the cavity length of approximately 19.3 m. A comparable pulse train was achieved by Zhou et al.^63^, where the slight fluctuation of the pulse peak intensities was affected by the relatively low sampling rate of the photodetector bandwidth and the low sampling points per pulse. Nonetheless, it was found that the pulse train's amplitude jitter was within reasonable limits. The radio frequency (RF) spectrum of the mode-locked pulse is shown in Fig. 11c, whereby a sharp peak was observed at about 10.73 MHz with a measured signal-to-noise ratio (SNR) of ~  55 dB. Figure 11d shows the autocorrelation trace of the mode-locked pulse, where the pulse width was measured to be 1.678 ps when fitted with a sech^2^ fitting. It was only about 19% longer than the calculated transform-limited pulse width. The corresponding TBP was 0.375, indicating that the pulse width was only slightly chirped.

**Figure 12:**
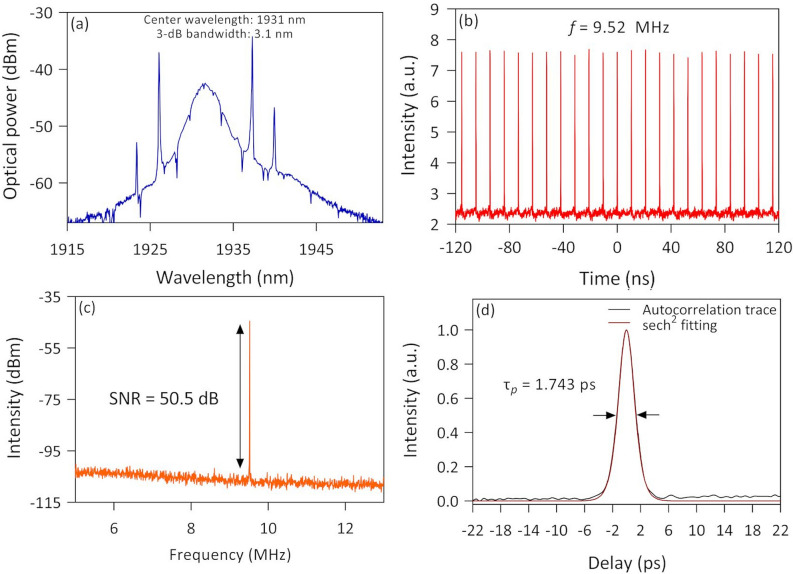
The output characteristic for mode-locked thulium-doped fiber laser using Ta_2_AlC side polished fiber saturable absorber (**a**) optical spectrum, (**b**) pulse train, (**c**) radio frequency spectrum, and (**d**) autocorrelation trace.

The experiment was then further conducted by replacing the Ta_2_AlC-deposited tapered fiber with the Ta_2_AlC-based side-polished fiber that has been described in Sect. 3.3. The fundamental mode-locking operation was achieved at a threshold pump power of 351 mW. The laser spectrum is given in Fig. 12a also shows a typical soliton spectrum with distinct Kelly's sidebands. However, it was observed that the sidebands were uneven, with the longer being higher than the shorter-wavelength sidebands. It is highly likely due to the optical fiber birefringence filtering effect in the cavity, as was theoretically and experimentally proven by Man et al.^80^. The center wavelength and the 3-dB bandwidth of the TDFL were recorded to be 1931 nm and 3.1 nm, respectively. As for the frequency of the mode-locked pulses, the oscilloscope trace shown in Fig. 12b shows a repetition rate of 9.52 MHz. The frequency was lower compared to the mode-locked laser with the Ta_2_AlC-deposited tapered fiber, which was due to the slightly longer length of the SPF. The RF spectrum of the mode-locked laser in Fig. 12c shows a sharp peak at around 9.52 MHz, having an SNR of ~ 50.5 dB. From the autocorrelation trace of the mode-locked pulse shown in Fig. 12d, the pulse width was measured to be 1.743 ps, fitted with a sech^2^ profile. The corresponding TBP was then calculated to be 0.434, also indicating that the pulse was chirped.

**Figure 13:**
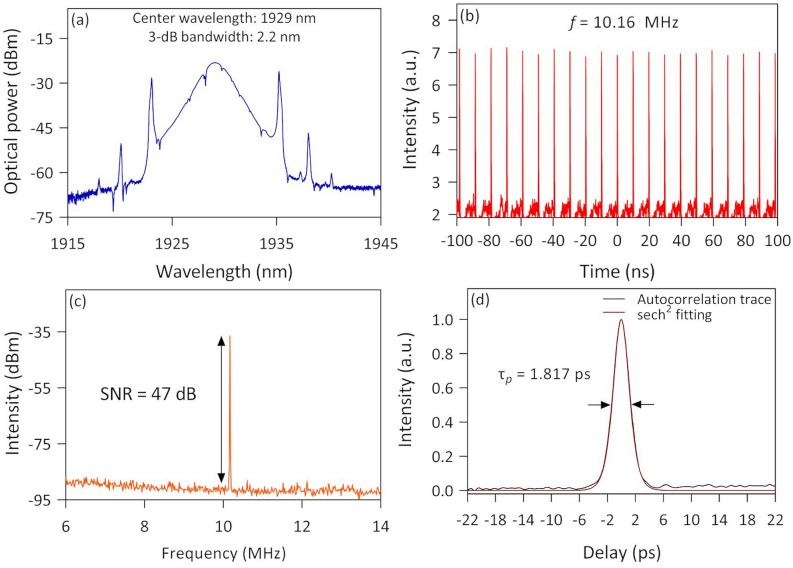
The output characteristic for mode-locked thulium-doped fiber laser using Ta_2_AlC arc-shaped fiber saturable absorber: (**a**) Optical spectrum, (**b**) soliton train, (**c**) radio frequency spectrum, and (**d**) autocorrelation trace.

The TDFL cavity was further tested by inserting the fabricated arc-shaped fiber with the Ta_2_AlC solution. The mode-locked pulses were generated at a pump power of 380 mW. The output characteristics of the mode-locked laser are shown in Fig. 13. From the optical spectrum plotted in Fig. 13a, the mode-locked laser had a center wavelength of 1929 nm with a 3-dB bandwidth of 2.2 nm. The presence of minor dips in the optical spectrum could be attributed to water absorption lines in 2 µm^81^. The mode-locked pulse had a repetition rate of 10.16 MHz with a 9.84 ns interval between peaks, measured from the oscilloscope trace in Fig. 13b. It tallies well with the fundamental frequency of the mode-locked laser, which was estimated by *f* = c/*n*L where c is the speed of light, *n* is the refractive index of an optical fiber, and L is the length of the cavity. By taking *n* to be approximately 1.44 at 2000 nm and L to be 20.43 m, the fundamental frequency was about 10.2 MHz. Figure 13c shows the RF spectrum with a peak that corresponds to the repetition rate of the mode-locked laser, in which it has an SNR value of ~ 47 dB. The autocorrelation trace of the pulse recorded using the autocorrelator is shown in Fig. 13d. When fitted with a sech^2^ fitting curve, the pulse width was measured to be 1.817 ps.

**Figure 14:**
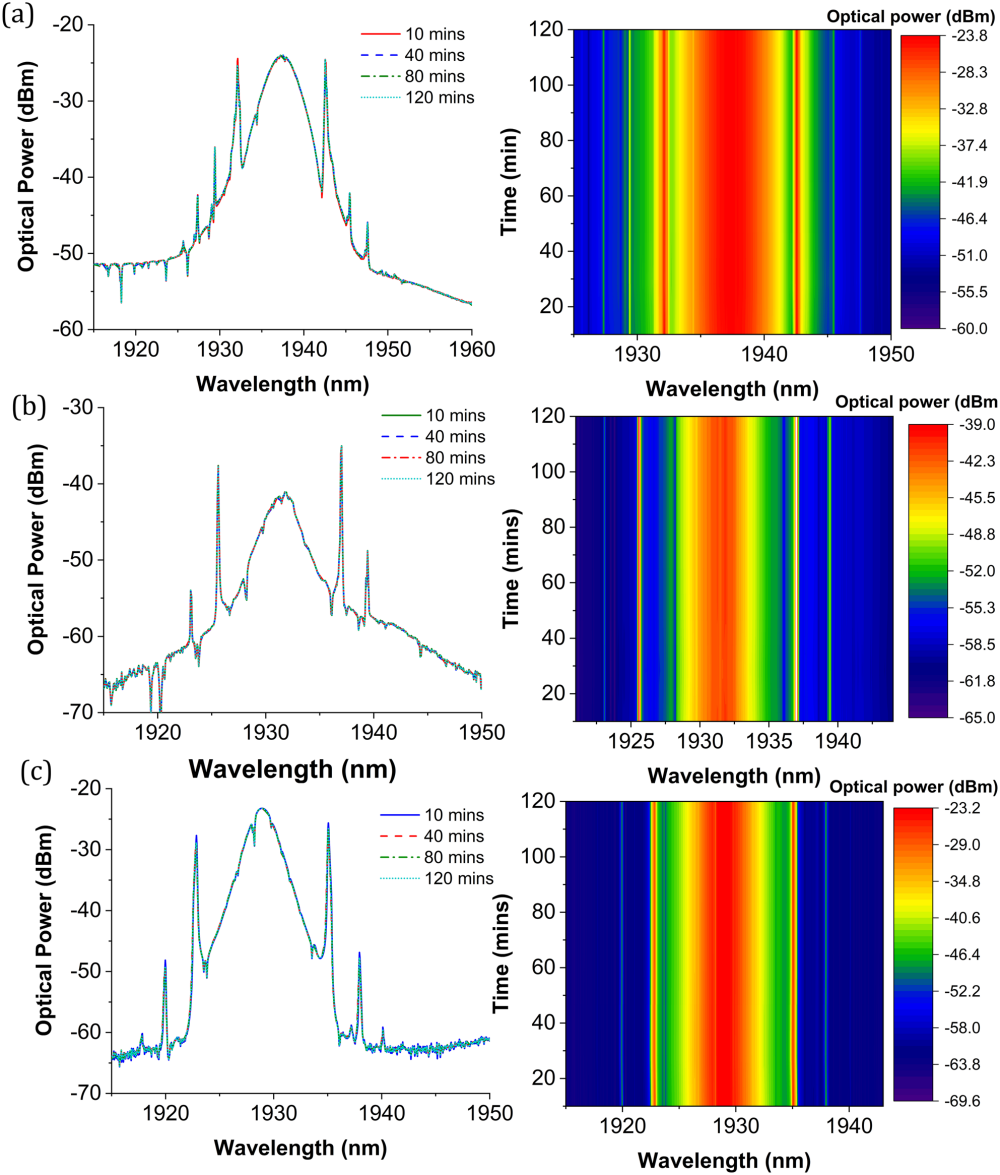
The stability of the output pulse spectrum for Ta_2_AlC and the corresponding contour plot for (**a**) tapered fiber, (**b**) SPF, and (**c**) arc-shaped fiber saturable absorber. The stability test was conducted for two hours at a 10-min interval.

The stability of the mode-locking operation with all three SA devices was evaluated by conducting a long-term stability test over two hours. The laser output for each of the three SA devices was monitored at every 10-min interval, in which their optical spectrum was recorded and plotted in Fig. 14. As seen from Fig. 14a, the mode-locked TDFL with the Ta_2_AlC-deposited tapered fiber exhibit a steady output intensity, and the contour plot shows no changes in the central wavelength of the output spectrum. For Ta_2_AlC-deposited SPF, the output spectrum displayed in Fig. 14b also shows a very stable output as the central wavelength and the Kelly sideband exhibit no changes throughout the stability test. Meanwhile, the arc-shaped fiber demonstrates a steady output as depicted in Fig. 14c.

**Figure 15:**
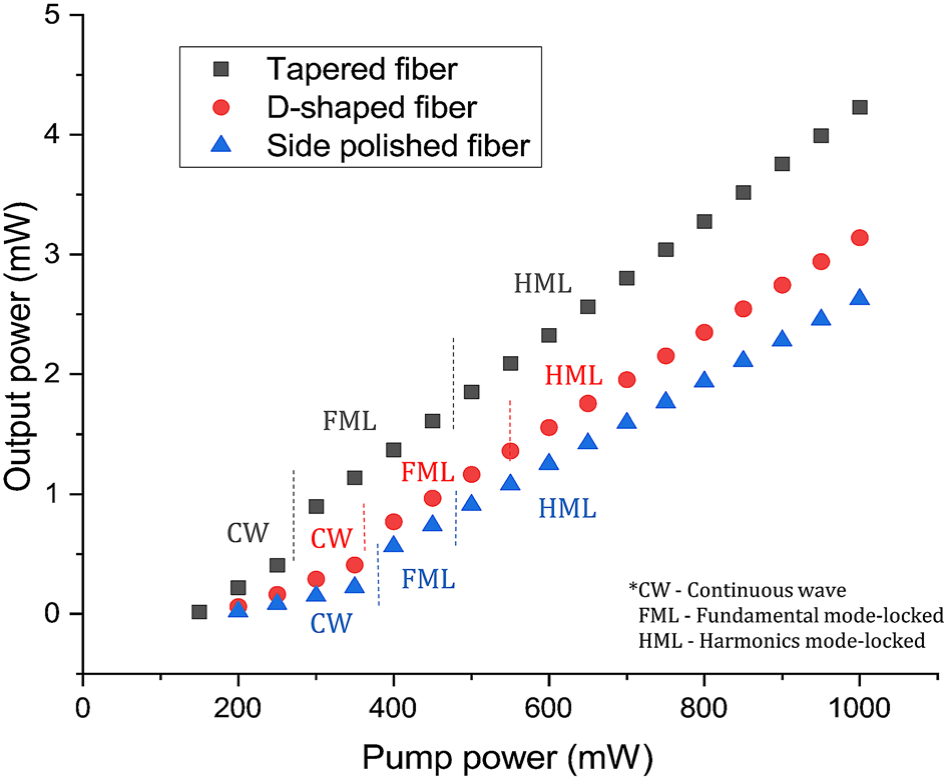
The pump power versus the output power of the mode-locked TDFLs.

The pump power against the output power of the mode-locked lasers is plotted in Fig. 15. As seen from the graph, the average output power of the laser with each type of SA device increased almost linearly after the mode-locking operation was initiated. The mode-locking threshold was 245 mW for the TDFL with the Ta_2_AlC-deposited tapered fiber, 351 mW for the Ta_2_AlC-deposited SPF, and 380 mW the Ta_2_AlC-deposited arc-shaped fiber. At this pump power, the fundamental mode-locking (FML) was observed. The FML operation was sustained until a pump power of 480 mW for the Ta_2_AlC-deposited tapered fiber and 479 mW and 550 mW for the Ta_2_AlC-deposited SPF and arc-shaped fiber, respectively. At the maximum pump power in which the FML was sustained, the average output power recorded for the mode-locked TDFL with the tapered fiber, SPF, side polished, and arc-shaped fiber was 1.91 mW, 0.8 mW 1.37 mW, respectively. When the pump power was further increased beyond these pump power, harmonic mode-locking was observed and could be maintained up until the maximum pump power of 1 W. At the maximum pump power of 1 W, the maximum average output power obtained were 3.48 mW, 2.27 mW and 2.71 mW for the TDFL with the tapered fiber, SPF and arc-shaped fiber, respectively. The TDFL with the Ta_2_AlC-deposited tapered fiber had the highest output power as the structure of the tapered fiber was maintained, only its dimension was reduced^82^.

In contrast, the mode-locked TDFL with the Ta_2_AlC-deposited SPF and arc-shaped fiber had a lower output power since the fiber structure had been modified during the grinding or polishing process. It caused a higher amount of light to escape easily by scattering light due to imperfection of the surfaces. Nevertheless, all the three SA devices could operate even when the pump power was increased until a maximum pump power of 1 W, without being optically damaged. Compared with materials embedded in polymer hosts, SAs in the film form typically had a lower damage threshold and can be easily burnt when the power was high^83^. It limits the application of polymer-based SAs in ultrafast fiber laser systems, eliminating the possibility of power-scaling of fiber lasers.

The performance of the mode-locked TDFLs with each of the three SA devices is summarized in Table 3.

**Table 3:** Summary of the optical characteristics of the TDFL with each mode-locking device in the fundamental operation.

Type of device	Threshold pump power (mW)	Repetition rate (MHz)	Signal to noise ratio (SNR)	Pulse width (ps)	Max. output power (mW)	Max. peak power (W)
Tapered	245	10.73	55	1.678	1.91	106
SPF	351	9.52	50.5	1.743	0.8	43
Arc-shaped	380	10.16	47	1.817	1.37	82

From Table 3, the lowest pump power needed to initiate the mode-locking operation was that of the Ta_2_AlC-deposited tapered fiber at 245 mW. This was followed by the Ta_2_AlC-deposited SPF and the arc-shaped fiber at the pump power of 351 mW and 380 mW, respectively. A low pump power threshold to induce the mode-locking operation was favorable as it could reduce the energy consumption. The mode-locked TDFL using the Ta_2_AlC-deposited tapered fiber generated the highest maximum average output power, which was as high as 3.48 mW. Compared to the output power of the TDFL using the Ta_2_AlC-deposited SPF and arc-shaped fiber, the generated output power was only 2.71 mW for the former and 2.27 mW for the latter. It was lower by about 22% from the output power generated by the TDFL with the Ta_2_AlC-deposited tapered fiber. The corresponding peak power could be calculated by dividing the average output power with the repetition rate and then dividing the value with the pulse width. It gives peak power values of 106 W, 43 W, and 82 W for the TDFL with the Ta_2_AlC-deposited tapered fiber, SPF, and arc-shaped fiber. The lowest and highest repetition rate recorded was 9.52 MHz with the Ti_2_AlC-deposited SPF and 10.73 MHz with the Ta_2_AlC-deposited tapered fiber. The difference in the repetition rate was only due to the length of the fiber used for the SA devices. In this regard, a higher repetition rate of the mode-locked TDFL could be obtained by having a shorter cavity length. The SNR values recorded from the RF spectrum for all three cases were more than 47 dB, which indicates that all three SA devices could generate stable mode-locked pulses. The shortest pulse width was recorded to be 1.678 ps, obtained using the Ta_2_AlC-deposited tapered fiber. The pulse widths obtained using the Ta_2_AlC-deposited SPF and arc-shaped fiber were slightly longer, with values of 1.734 ps and 1.817 ps being measured. Overall, it is seen that all the SA devices could generate mode-locked pulses in the 2 µm region, with the Ta_2_AlC-deposited tapered fiber providing the best performance in terms of the low mode-locking threshold, the highest output, and peak power, as well as having the shortest pulse width. It is noted that the shorter pulse width obtained could be attributed to the cavity with the Ta_2_AlC-deposited tapered fiber working with the lowest anomalous net cavity dispersion. This was further confirmed by Zhang et al.^84^, who demonstrated that when the anomalous dispersion value decreased, the pulse width value also decreased proportionally. Another contributing factor to the better performance could also be due to the tapered fiber being able to maintain a circular and symmetrical structure, compared to the side-polished and arc-shaped fibers having unsymmetrical fiber structures after the polishing process. Thus, the results show the potential of tapered fibers to be further explored for the development of high-power ultrafast fiber lasers.

## Methods

### Preparation of Ta_2_AlC MAX phase

The Ta_2_AlC was purchased from Forsman Scientific (Beijing) Co., Ltd in powder form with a purity of ≥ 98%. It was prepared directly by dissolving 30 mg of Ta_2_AlC powder in 3 ml isopropyl alcohol (IPA) and then sonicated for 1 h.

### Laser characterization

The output characteristics of the laser were obtained using a Rohde & Schwarz RTM3002 oscilloscope (OSC), Yokogawa AQ6375 optical spectrum analyzer (OSA), Rohde & Schwarz FPC1000 radio frequency spectrum analyzer (RFSA), and an A.P.E. PulseCheck150 optical autocorrelator.

## Conclusion

Mode-locked thulium-doped fiber lasers (TDFLs) operating in the 2 μm were successfully demonstrated using three different SA devices: tapered fiber, side-polished fiber (SPF), and also the arc-shaped fiber. The tapered fiber was fabricated using the flame-brushing method, while the arc-shaped fiber was polished using the wheel-polishing technique. Meanwhile, the SPF was obtained commercially. A new type of MAX phase, the Ta_2_AlC, was drop-casted onto the three fibers and then inserted separately into the TDFL cavity. The MAX phase was composed of tantalum (Ta) as the early transition metal instead of the common titanium (Ti). Stable mode-locked pulses were obtained for all cases using the three SA devices. The stability measurements showed little to no fluctuations in the center wavelength and the peak optical power of the mode-locked TDFLs. The SNR values were also recorded to be more than 47 dB for all three cases, further proving the stability of the generated mode-locked pulses. It was observed from the results that the Ta_2_AlC-deposited fiber had the best performance as it can generate the highest output power of 1.91 mW, the highest peak power of 106 W, and had the shortest pulse width of 1.678 ps. These demonstrations show the potential of SA devices that utilize the evanescent field interaction to be used as SAs for the development of high-power ultrafast fiber lasers.
